# Corrigendum

**DOI:** 10.1080/19491034.2018.1468402

**Published:** 2018-05-10

Title: “A demethylation deficient isoform of the lysine demethylase KDM2A interacts with pericentromeric heterochromatin in an HP1a-dependent manner”

Author name: Dijana Lađinović, Jitka Novotná, Soňa Jakšová, Ivan Raška, Tomáš Vacík

**Journal:** Nucleus

**Bibliometrics:** Volume 8, Issue 5, pages 563–572

**DOI:** 10.1080/19491034.2017.1342915

The Authors wish to make changes to the listed affiliations and funding:

Dijana Lađinović^a^, Jitka Novotná^a^, Soňa Jakšová^a^, Ivan Raška^a^, Tomáš Vacík^a,b^

^a^Institute of Biology and Medical Genetics, First Faculty of Medicine, Charles University and General University Hospital in Prague, Prague, Czech Republic; ^b^Institute of Molecular Genetics of the Academy of Sciences of the Czech Republic, v.v.i., 142 20, Prague, Czech Republic

This work was supported by the Czech Science Foundation grant P302/12/G157, by the Charles University grants UNCE 204022 and PRVOUK P27/LF1/1, and by the OPPK grant CZ.2.16/3.1.00/24010. This work was also supported by the Czech Science Foundation grant 15–08738S and the following grants: Institutional Research Concept of the Institute of Molecular Biology RVO: 68378050 and the MEYS CR LM2015062 Czech BioImaging.

The Authors apologize for inconvenience.

